# The effect of hypoxia on the lipidome of recombinant *Pichia pastoris*

**DOI:** 10.1186/s12934-017-0699-4

**Published:** 2017-05-19

**Authors:** Núria Adelantado, Pablo Tarazona, Karlheinz Grillitsch, Xavier García-Ortega, Sergi Monforte, Francisco Valero, Ivo Feussner, Günther Daum, Pau Ferrer

**Affiliations:** 1 Department of Chemical, Biological and Environmental Engineering, Escola d’Enginyeria, Universitat Autònoma de Barcelona, Bellaterra, 08193 Barcelona, Catalonia Spain; 2 Department of Plant Biochemistry, Albrecht-von-Haller-Institute for Plant Sciences, Georg-August-University, Justus-von-Liebig-Weg 11, 37077 Göttingen, Germany; 3 Department of Plant Biochemistry, Göttingen Center for Molecular Biosciences (GZMB), Georg-August-University, Justus-von-Liebig-Weg 11, 37077 Göttingen, Germany; 4 Austrian Centre of Industrial Biotechnology (ACIB), Graz, Austria; 5 Institute of Biochemistry, Graz University of Technology, Petersgasse 12/II, 8010 Graz, Austria; 6 Evonik Nutrition & Care GmbH, Hanau, Germany

**Keywords:** Lipidomics, *Pichia pastoris*, Hypoxia, Recombinant protein production, Antibody fragment, Protein secretion, Unfolded protein response

## Abstract

**Background:**

Cultivation of recombinant *Pichia pastoris* (*Komagataella* sp.) under hypoxic conditions has a strong positive effect on specific productivity when the glycolytic *GAP* promoter is used for recombinant protein expression, mainly due to upregulation of glycolytic conditions. In addition, transcriptomic analyses of hypoxic *P. pastoris* pointed out important regulation of lipid metabolism and unfolded protein response (UPR). Notably, UPR that plays a role in the regulation of lipid metabolism, amino acid metabolism and protein secretion, was found to be upregulated under hypoxia.

**Results:**

To improve our understanding of the interplay between lipid metabolism, UPR and protein secretion, the lipidome of a *P. pastoris* strain producing an antibody fragment was studied under hypoxic conditions. Furthermore, lipid composition analyses were combined with previously available transcriptomic datasets to further understand the impact of hypoxia on lipid metabolism. Chemostat cultures operated under glucose-limiting conditions under normoxic and hypoxic conditions were analyzed in terms of intra/extracellular product distribution and lipid composition. Integrated analysis of lipidome and transcriptome datasets allowed us to demonstrate an important remodeling of the lipid metabolism under limited oxygen availability. Additionally, cells with reduced amounts of ergosterol through fluconazole treatment were also included in the study to observe the impact on protein secretion and its lipid composition.

**Conclusions:**

Our results show that cells adjust their membrane composition in response to oxygen limitation mainly by changing their sterol and sphingolipid composition. Although fluconazole treatment results a different lipidome profile than hypoxia, both conditions result in higher recombinant protein secretion levels.

**Electronic supplementary material:**

The online version of this article (doi:10.1186/s12934-017-0699-4) contains supplementary material, which is available to authorized users.

## Background

The methylotrophic yeast *Pichia pastoris* (*Komagataella* sp.) has become an important cell factory for heterologous protein production [1–3]. *P. pastoris* is a eukaryote, and therefore provides the potential for producing soluble, correctly folded recombinant proteins that have undergone all post-translational modifications required for functionality. Furthermore, this yeast can be engineered to mimic the human *N*-glycosylation pathway and specific types of *O*-glycosylation, becoming a potential alternative for mammalian cell culture for the production of recombinant therapeutic glycoproteins for human use [4, 5]. Overexpression of heterologous proteins can lead to saturation or overloading of the secretory pathway [6, 7]. The most important bottlenecks in terms of recombinant protein production and secretion are membrane translocation, signal peptide processing and folding within the endoplasmic reticulum (ER) [8]. Strain engineering strategies for protein secretion are mainly focused on engineering the protein folding and quality control systems in the ER, the intracellular protein trafficking pathway, and minimizing post-secretory degradation [9]. In addition, there is increasing evidence that metabolic bottlenecks in the supply chain for building blocks and energy play an important role in recombinant yeast [10, 11]. In this context, environmental conditions have a significant impact on the levels of recombinant proteins. For instance, lower culture temperature [12, 13], low oxygen availability [14], as well as adequate substrate feeding strategies in high cell density cultures [15, 16], and type of carbon source(s) [17, 18] resulted in positive effects on protein secretion.

Oxygen availability is critical for many biochemical reactions in eukaryotic cells, including yeasts. The ability to adapt to oxygen limitation is essential for cell survival but also produces important metabolic, functional and structural changes in the cell [19]. Cells can adapt to growth under oxygen limitation, termed hypoxia or microaerobic conditions [20]. When cells are grown aerobically, molecular oxygen serves as the final electron acceptor for respiration, while it is also used for the synthesis of metabolites, e.g. sterols or unsaturated fatty acids. In the presence of low amounts of oxygen, respiration is drastically reduced, and metabolism is then reprogrammed to optimize yeast cells for fermentative dissimilation of the carbon source to conserve energy and to maintain a closed redox balance [21]. These metabolic rearrangements are easily detectable by the excretion of metabolites in the culture supernatant such as ethanol and arabitol in the case of *P. pastoris* [22].

The impact of oxygen limitation on recombinant protein production in *P. pastoris* was first studied by Baumann and co-workers [14] showing a significant increase of the specific production rate of several model recombinant proteins. In a subsequent study, the impact of oxygen availability on the physiology of recombinant *P. pastoris* was studied integrating transcriptomic, proteomic, metabolic flux and metabolomics analyses [22–24]. In response to oxygen limitation, a wide range of transcriptional modifications occurred, resulting in extensive changes of cellular protein levels and activities, including those related to cell respiration, lipid metabolism, cell membrane and cell wall structure [23, 25]. Increased transcript levels were observed for a number of genes encoding enzymes that catalyze oxygen-consuming reactions of the ergosterol pathway (*ERG1*, *ERG3*, *ERG5*, *ERG11* and *ERG25*). Similarly, expression of sphingolipid synthesis genes (*SUR2*, *SCS7*, *DES1* and *SLD1*) was also upregulated under hypoxic conditions, as all these enzymes need molecular oxygen as substrate [23]. Notably, overexpression of the unfolded protein response (UPR) genes such as *HAC1*, *PDI1*, *ERO1* and *HAC1* as also detected in hypoxia. Changes observed on lipid metabolic enzymes affect lipid composition of the membrane such as fluidity [26, 27] and other physiological traits [28–30], some of which could ultimately favor recombinant protein secretion. Indeed, altered activity of the lanosterol C-14α demethylase (*ERG11*), which catalyzes a rate-limiting step in ergosterol biosynthesis [31], by treating cells with the antifungal agent fluconazole results in lower ergosterol levels and increased Fab secretion (1.4-fold) compared to untreated cells [32]. Such changes in the total sterol content of membranes might result in increased membrane fluidity and higher levels of protein secretion. Moreover, cultivation in the presence of non-ionic surfactants such as Tween 20, Tween 80 and Triton X-100 also resulted in increased levels of secreted Fab (up to 1.65-fold), probably due to a similar effect, i.e. higher membrane fluidity when cells were grown in the presence of these surfactants.

Lipid composition of *P. pastoris* organelles such as the plasma membrane [33], peroxisomes [34], mitochondria [35], lipid droplets [36], or endoplasmic reticulum [37] have already been characterized. The lipid composition of this yeast has also been studied with regard to a carbon source effect [38]. However, such fundamental studies were performed exclusively using wild type strains.

In the present study, we describe the biochemical characterization of *P. pastoris* lipidome after adaptation to hypoxia. This analysis is based on the characterization of lipids from a *P. pastoris* strain producing a recombinant antibody fragment (Fab) grown under oxygen-excess (normoxic) and reduced oxygen availability (hypoxic) conditions in chemostat cultures, where well-controlled and reproducible culture conditions are provided. Changes in the lipidome were correlated with corresponding transcriptional changes reported for this condition in earlier studies [23], which were further verified by quantitative PCR in this study. Moreover, cells treated with fluconazole were analyzed to elucidate whether the observed increase in Fab secretion was correlated with similar changes in lipid composition (beyond reduced ergosterol levels) as under hypoxic conditions.

The aim of the work was to expand our knowledge of *P. pastoris* lipid metabolism adaptation to hypoxia and the implications for recombinant production by identifying changes in lipid composition that appear to be correlated with the improvement of protein secretion in hypoxic culture conditions. Moreover, through combined measurements of the transcriptome and lipidome it was possible to identify the effect of hypoxia on other cellular processes related to lipid metabolism such as UPR, thereby verifying interrelations between the different processes and protein secretion.

## Results and discussion

### Hypoxia and fluconazole treatment do not exert synergistic effects on protein secretion

Previous studies cultivating *P. pastoris* under hypoxic chemostat conditions revealed that stringent hypoxia leads to bioreactor wash out [14]. To establish less severe working hypoxic conditions, which still result in respirofermentative metabolism but prevent culture instability, *P. pastoris* producing Fab 2F5 was grown in glucose-limited chemostat cultures using different concentrations of oxygen in the inlet gas. The desired working hypoxic condition was defined as the lower air flow that permitted a stable cell concentration, i.e. no washout in the bioreactor while significant amounts of ethanol and arabitol were present in the media, thereby indicating respirofermentative metabolic condition. Based on this preliminary series of chemostat experiments, permissive hypoxic conditions were established as defined in “Methods”.

Subsequently, a series of carbon-limited chemostat cultivations at a growth rate of 0.1 h^−1^ were performed. Cells were cultured under normal oxygen conditions (normoxia) and defined low oxygen conditions (hypoxia). To further explore the effect of hypoxia on lipid composition and its potential impact on protein secretion, the same series of chemostat cultivations were also carried out in the presence of fluconazole in the growth medium, which was reported in previous studies to have beneficial effects on protein secretion [32]. The appropriate fluconazole concentration was established in relation to cell mass allowing for the maximal protein secretion without compromising cell growth (see “Methods”). Cultivations were analyzed in terms of biomass and specific Fab productivity (Table 1). As expected, ethanol and arabitol were detected in the culture medium of hypoxic cultivations, biomass yield was reduced and respiratory quotient (RQ) was increased, indicating that cells were growing under respirofermentative conditions. Ethanol and arabitol specific production rates were lower than in previous hypoxic studies [22], pointing out less stringent hypoxic stress and providing true steady state conditions in the bioreactor (i.e. no wash out).

**Table 1:** Summary of the macroscopic culture parameters

Culture conditions	DCW (g L^−1^)	Y_X/S_ (g_DCW_ ${\text{g}}_{\text{glc}}^{ - 1}$)	Fab secreted (mg_Fab_ L^−1^)	q_Fab_ (mg_Fab_ ${\text{g}}_{\text{DCW}}^{ - 1}$ h^−1^)	q_EtOH_ (mmol_EtOH_ ${\text{g}}_{\text{DCW}}^{ - 1}$ h^−1^)	q_Ara_ (mmol_Ara_ ${\text{g}}_{\text{DCW}}^{ - 1}$ h^−1^)	q_O2_ (mmol_O2_ ${\text{g}}_{\text{DCW}}^{ - 1}$ h^−1^)	q_CO2_ (mmol_CO2_ ${\text{g}}_{\text{DCW}}^{ - 1}$ h^−1^)	RQ	C-balance
Normoxia	28.5 ± 0.0	0.58 ± 0.00	5.0 ± 0.2	0.017 ± 0.008	0.001 ± 0.000	n.d.	2.04 ± 0.01	2.00 ± 0.01	1.0 ± 0.0	1.02
Hypoxia	20.5 ± 0.4	0.42 ± 0.00	11.0 ± 0.1*	0.049 ± 0.012*	0.434 ± 0.058	0.048 ± 0.013	1.89 ± 0.05	2.60 ± 0.05	1.4 ± 0.0	0.97
Fluconazole	25.0 ± 0.0	0.50 ± 0.01	5.6 ± 0.1*	0.021 ± 0.004	n.d.	0.001 ± 0.000	2.25 ± 0.05	2.57 ± 0.05	1.1 ± 0.0	0.98
Fluconazole + hypoxia	8.9 ± 0.0	0.28 ± 0.00	3.0 ± 0.1*^†^	0.033 ± 0.014	1.181 ± 0.017	0.456 ± 0.009	3.74 ± 0.07	5.60 ± 0.10	1.5 ± 0.0	1.18

Physiological parameters of the *P. pastoris* strain producing Fab 2F5 grown in normoxic and hypoxic conditions, in the presence or absence of fluconazole, in glucose-limited chemostat cultures at D = 0.1 h^−1^. Values represent the mean ± SD

*DCW* dry cell weight, *glc* glucose, *Y*_*X/S*_ biomass to substrate yield, *q* specific consumption/product formation rates, *RQ* respiratory quotient, *C-balance* carbon balance, *n.d.* not detected. Glucose and glycerol where measured by HPLC and values were always under the detection level

* p < 0.05 for the *t* tests compared to the normoxia culture

^†^ p < 0.05 for the *t* tests compared to fluconazole culture

Specific Fab production rate in hypoxia was 2.9-fold higher than in normoxia, while fluconazole treatment increased protein secretion by 1.24-fold compared to normoxic conditions. These results were coherent with previously reported findings [14, 32], which revealed increased transcriptional levels from the glycolytic GAP promoter used to drive recombinant protein expression in *P. pastoris*, as well as increased protein secretion upon fluconazole treatment. Conversely, rather than a synergistic effect, fluconazole treatment of hypoxic cultures resulted only in a 1.9-fold increased Fab productivity in relation to the reference normoxic condition, probably due to the additive effects of hypoxia and fluconazole impairing de novo sterol synthesis. Additionally, lower biomass yield under hypoxia resulted in a higher fluconazole to biomass ratio, i.e. different from the optimum established for highest Fab secretion in shake flask experiments. In fact, these conditions led to a pseudo-steady state that ended up in the washout of the reactor after five residence times.

### High Fab secretion yield is observed in all tested culture conditions

The amount of Fab present in the extracellular fraction (i.e. secreted Fab), soluble cytosolic fraction and insoluble membrane fraction were quantified for all culture conditions. Thus, intracellular and extracellular distribution of the Fab within the cells were determined (Table 2). Under all conditions tested, the relative secretion levels of Fab were above 85%, that is increased secretion levels observed in hypoxia were not accompanied by higher intracellular Fab levels. Notably, the insoluble fraction, i.e. the membrane fractions of the cell including ER, plasma membrane contained almost no recombinant protein, indicating no intracellular protein accumulation in the ER due to misfolding/aggregation events or retention in the periplasmic space. Nevertheless, we cannot exclude the possibility that misfolded/aggregated Fab was efficiently removed by the ERAD pathway as reported previously [39].

**Table 2:** Distribution of the produced Fab 2F5

Culture conditions	D (h^−1^)	q_Fab_ (mg_Fab_ ${\text{g}}_{\text{DCW}}^{ - 1}$ h^−1^)	Fab expression (mg_Fab_ ${\text{g}}_{\text{DCW}}^{ - 1}$)				%
			Extracellular fraction	Cytosolic fraction	Membrane fraction	Total	Secretion
Normoxia	0.094 ± 0.005	0.017 ± 0.008	0.254 ± 0.017	0.023 ± 0.001	0.005 ± 0.000	0.282 ± 0.017	90.1 ± 4.7
Hypoxia	0.092 ± 0.005	0.049 ± 0.012*	0.534 ± 0.007*	0.033 ± 0.002*	0.005 ± 0.001	0.572 ± 0.007	93.4 ± 1.6
Fluconazole	0.094 ± 0.006	0.021 ± 0.004	0.223 ± 0.004*	0.024 ± 0.002	0.007 ± 0.001*	0.254 ± 0.004	87.8 ± 2.1
Fluconazole + hypoxia	0.100 ± 0.006	0.033 ± 0.014	0.333 ± 0.015*^†^	0.039 ± 0.002*^†^	0.012 ± 0.001*^†^	0.384 ± 0.015	86.8 ± 5.3

Values represent the mean ± SD

*D* dilution rate, *q*_*Fab*_ specific product formation rate

* p < 0.05 for the *t* tests compared to the normoxia culture

^†^ p < 0.05 for the *t* tests compared to fluconazole culture

Although secretion of heterologous proteins is liable to several bottlenecks that limit yield [6], these results suggest that despite an increase in Fab synthesis under the selected hypoxic conditions, this was not enough to result in a stronger secretion limitation. This may indicate that membrane alterations due to hypoxic culture conditions favored protein secretion, avoiding intracellular accumulation even when Fab production was increased.

### Integrated transcriptomic-lipidomic analysis of the hypoxia effect

**Fig. 1:**
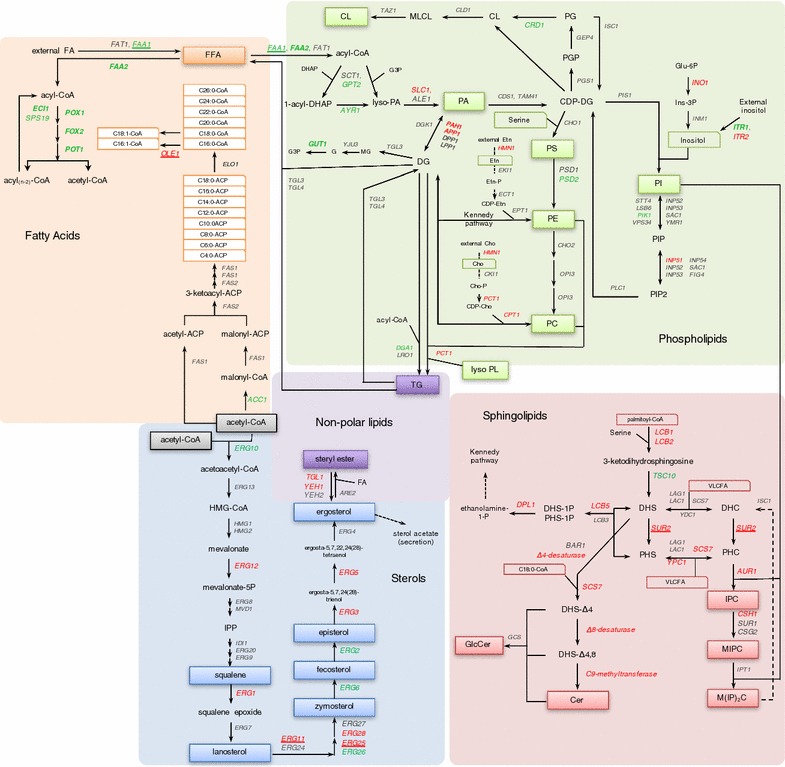
Schematic representation of lipid biosynthesis pathways from *P. pastoris* and its regulation in hypoxia. Genes under hypoxic conditions were compared to normoxic conditions. Lipid species analyzed in the study are boxed, and genes selected to perform transcriptional analysis by quantitative PCR (ddPCR) are *underlined*. Fold changes of genes are indicated by color: *red* upregulated genes, *green* downregulated genes, *grey* no significant changes. (Based on *p* values <0.05). Transcriptional data was taken from [23]

Lipid composition of cell homogenates was determined for cells growing under normoxic and hypoxic conditions. Previously published transcriptomic datasets for hypoxic conditions (Fig. 1) [23] were used together with the lipid profile alterations resulting from this culture condition.

#### Changes in fatty acid unsaturation were correlated with transcriptional changes of OLE1 encoding fatty acid desaturase

**Fig. 2:**
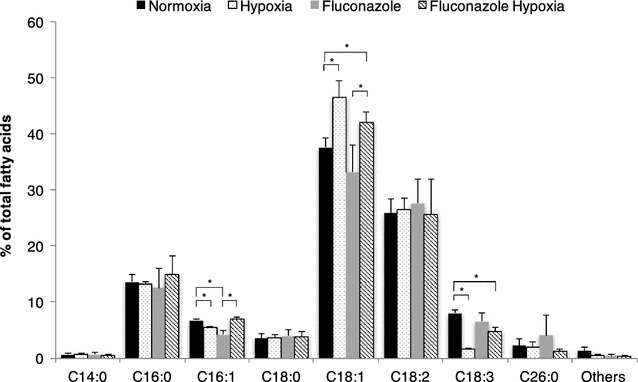
Cellular fatty acid composition. Fatty acid composition (% of total) of *P. pastoris* cells producing the Fab 2F5 and growing under normoxic or hypoxic conditions in the presence or absence of fluconazole. Data represent mean values ± SD from triplicates. *p < 0.05 for the *t* tests

Relative amounts of fatty acids in cells were analyzed for different growth conditions. Under hypoxia, a change in the degree of unsaturation was given by a significant increment of oleic acid (C18:1n-9, *x*:*y*n-*z* denotes a fatty acid with *x* carbons and *y* double bonds in position *z* counting from the methyl end) and a decrease of α-linolenic acid (C18:3n-3) species present in the cells (Fig. 2). Previous transcriptional analysis of the reference strain cultivated under hypoxic conditions indicated an upregulation of the O_2_-dependent Δ^9^-fatty acid desaturase *OLE1* (Fig. 1); this has been further confirmed by ddPCR for the Fab2F5-producing strain, showing a 1.4-fold increase under hypoxia. Ole1p is involved in the biosynthesis of unsaturated fatty acids. In *Saccharomyces cerevisiae, OLE1* gene is highly regulated in response to various environmental signals such as low temperature and hypoxic conditions through the sensor Mga2p [40, 41], and to unsaturated fatty acid concentration through Spt23p. However, lack of *SPT23* in *P. pastoris* may indicate that this microorganism possesses an alternative gene for unsaturated fatty acid sensing, or it only regulates fatty acid unsaturation through *MGA2.* Hence, reduced oxygen availability could explain the increase in the relative amounts of monounsaturated fatty acid, while the more energy and oxygen demanding generation of di- and tri-unsaturated fatty acids would be reduced [42]. The presence of oleic acid, a monounsaturated fatty acid, as the major fatty acid component, but not saturated fatty acids, may suggest that oleic acid plays a central role in maintaining membrane fluidity and, modulating protein secretion under hypoxia.

#### Changes in the phospholipid pattern correlate with the presence/absence of intracellular levels of free inositol and UPR upregulation under hypoxia

**Fig. 3:**
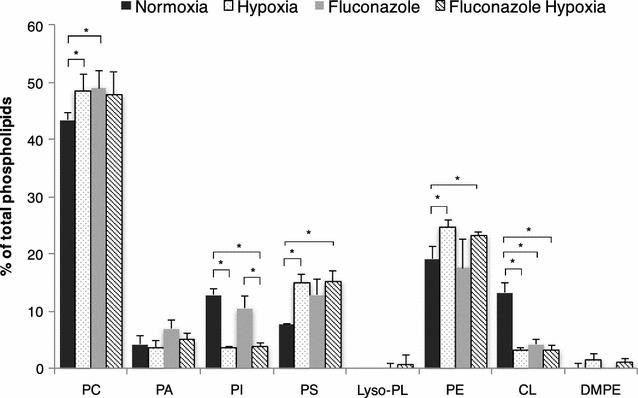
Cellular phospholipid composition. Phospholipid composition (% of total phospholipids) of the cells growing under normoxic or hypoxic conditions, in the presence or absence of fluconazole. *PC* phosphatidylcholine, *PA* phosphatidic acid, *PI* phosphatidylinositol, *PS* phosphatidylserine, *Lyso-PL* lysophospholipids, *PE* phosphatidylethanolamine, *CL* cardiolipin, *DMPE* dimethyl phosphatidylethanolamine. Data represent mean values ± SD from duplicates. *p < 0.05 for the *t* tests comparing phospholipid detected values

The most significant changes in phospholipid patterns observed as a result of hypoxic conditions were the significant increment of phosphatidylserine (PS), while phosphatidylinositol (PI) levels dropped in cells grown under this cultivation condition (Fig. 3). PI and PS are synthesized by Pis1p and Cho1p, respectively, which compete for CDP-DG, making this metabolic branch an important point of regulation [43]. However, no significant changes at the transcriptomic level were observed for these two genes under hypoxia (Fig. 1). PS and PI are key determinants of membrane surface charge. Both types of phospholipids are anionic (charge -1), but they differ in their shape. PS is cylindrical shaped and preferentially forms flat bilayer structures, while PI has an inverted conical shape and forms structures with positive curvatures [44]. Uneven distribution of PS and PI causes variation of the electrostatic properties of the membrane creating as an example a highly charged cytosolic leaflet on the plasma membrane [45]. Membrane deforming domains are crucial for protein membrane interactions. Moreover, some domains and proteins prefer PI rather than PS as interaction partners, making PI a major player on controlling a variety of cellular functions [46]. Thus, changes of the relative amounts of PI observed in cells growing under hypoxia can result in alterations of membrane interactions and affect some cellular functions.

Inositol is a precursor of PI [47], and also a potent regulator of phospholipid metabolism in yeast. Inositol used in PI synthesis is either synthesized *de novo* through *INO1*, or obtained from the growth medium via the *ITR1*- and *ITR2*-encoded inositol transporters [43]. Our transcriptional dataset indicated that *ITR1* transcript levels were highly downregulated under hypoxic conditions, while *ITR2* and *INO1* were upregulated (Fig. 1). When the intracellular amount of inositol decreases, the level of *OPI1*, a negative regulator of a large number of phospholipid biosynthetic genes, is also reduced, favoring transcription of a large variety of genes containing the “inositol-sensitive upstream activating sequence” (UAS_INO_) [48]. Furthermore, phosphatidic acid, a precursor of most phospholipids, is also an important regulator of the *OPI1* level within the cell [47]. In *S. cerevisiae,* Opi1p represses UAS_INO_ genes through direct interaction with the heterodimer Ino2p-Ino4p [49], while regulation of this biosynthetic pathway in *P. pastoris* is still unclear due to the lack of *INO2*. Our transcriptomic data indicate a downregulation of the *OPI1* levels. However, only the UAS_INO_ gene *INO1 was* upregulated under hypoxia, while no significant changes in other genes containing the UAS_INO_ element were observed. Furthermore, relative amounts of PS and PE increased under hypoxia (Fig. 3). The observed changes in the phospholipid pattern may be related to the decreased amount of inositol availability in cells growing under hypoxic conditions. It is known that cells growing in the absence of inositol contain a low PI content that may result in UPR pathway activation [50]. Moreover, the transcriptional factor Hac1p, mediates the activation of the UPR, negatively regulates the activity of Opi1p and, in turn, it also plays a role in the regulation of phospholipid biosynthesis [51]. Under hypoxic conditions, *HAC1* was upregulated, thus favoring UPR [23]. Moreover, UPR upregulation by hypoxia has been further confirmed in this study by measuring transcriptional levels of *HAC1*, *ERO1* and *PDI1* genes by ddPCR, which were increased 3-, 2.5- and 2-fold, respectively, under hypoxia. High levels of Hac1p may result in the upregulation of *INO1* [51]. Subsequently, the UPR pathway could be part of a generalized stress response occurring when cells are deficient in inositol [52].

Accumulation of misfolded proteins in the ER activates the UPR [1, 53–55]. Such response is mediated by Ire1p. In addition, Ire1p can also sense lipid stress through an alternative activation process [56]. In particular, Ire1p senses changes in the biophysical properties of membranes by sensing the ratio of unsaturated to saturated acyl chains through their transmembrane domains [57] and it also responds to low inositol levels activating UPR [51]. Furthermore, Ire1p is required for the expression of *INO1* in the absence of exogenous inositol [58]. As our data suggest that hypoxic conditions alter lipid composition of the cells, mainly through the oxygen-dependent reactions (fatty acid desaturation, ergosterol and sphingolipid biosynthesis) and drop of inositol levels, these changes may be sensed by Ire1p, thereby activating the UPR, affecting lipid metabolism, membrane biogenesis and protein secretion.

#### Ergosterol content is reduced due to hypoxic conditions

Ergosterol is a component required to maintain membrane integrity and it is essential for cell viability [59]. Under hypoxic conditions, genes of the ergosterol pathway were highly upregulated (Fig. 1), correlated with a tendency for reduced ergosterol content (Table 3). Coherently, transcriptional analysis by ddPCR of *ERG11* and *ERG25* genes for the Fab2H5-producing strain show about 2.6- and 2.1-fold increase under hypoxia, respectively. However, no regulation at the transcriptomic level was observed for the transcription factor Upc2p, responsible for the transcriptional activation of genes involved in the sterol biosynthetic pathway [60]. Sharma [27] suggested an adaptive response to altered sterol structures through changes in the lipid composition and fluidity that could occur upon sterol deprivation. For instance, yeast cells adjust their sphingolipid content in response to changes in ergosterol content, which in turn may result into changes of the entire lipid composition [26, 61] leading to a beneficial effect on protein secretion, as it will be further discussed below.

**Table 3:** Cellular sterol composition

Culture conditions	µg sterol/mg total protein							
	Squalene	Lanosterol	Ergostadienol	4-Methyl zymosterol	Zymosterol	Fecosterol	Episterol	Ergosterol
Normoxia	n.d.	n.d.	n.d.	0.89 ± 0.36	0.75 ± 0.44	n.d.	0.17 ± 0.05	8.84 ± 1.98
Hypoxia	n.d.	0.14 ± 0.00	n.d.	0.80 ± 0.18	0.52 ± 0.23	n.d.	0.38 ± 0.19	6.21 ± 1.20
Fluconazole	n.d.	1.39 ± 0.51*	0.21 ± 0.06	n.d.	0.10 ± 0.05	0.78 ± 0.39	0.18 ± 0.05	6.43 ± 1.24
Fluconazole + hypoxia	0.18 ± 0.07	5.06 ± 0.57*^†^	0.77 ± 0.35	n.d.	0.58 ± 0.41	0.90 ± 0.25	0.94 ± 0.47*^†^	7.71 ± 1.54

Sterol composition of cells growing under normoxic or hypoxic conditions, in the presence or absence of fluconazole

*n.d.* not detectable. Values represent the mean ± SD of triplicates

* p < 0.05 for the *t* tests compared to the normoxia culture

^†^ p < 0.05 for the *t* tests compared to fluconazole culture

#### Non-polar lipids accumulate under hypoxic conditions due to inositol depletion

The regulatory interplay and metabolic interrelation between storage lipids, i.e. triacylglycerols (TG), and membrane lipids, i.e. phospholipids, have been recognized as an important determinant of cellular growth and proliferation in *S. cerevisiae* [62, 63]. Hypoxic conditions resulted in significantly high levels of TG (Table 4). TG synthesis from phosphatidic acid by the action of Pah1p, upregulated in hypoxia, may be trigged by low levels of inositol in *S. cerevisiae* [64]. Moreover, it has been reported that changes in glucose metabolism caused by the shift from respiratory to respirofermentative metabolism can also affect non-polar lipid homeostasis in this yeast by changing activity of TG lipases [65]. The enzymes Nte1p and Lro1p, which turned out to be upregulated in hypoxia, catalyze reactions that either directly or indirectly promote synthesis of TG and contribute to the adjustment of the composition of membrane phospholipids [65].

**Table 4:** Neutral lipid composition

Culture conditions	µg lipid/mg total protein	
	TG	SE
Normoxia	92.9 ± 26.0	6.89 ± 1.40
Hypoxia	156.4 ± 32.6	9.38 ± 0.50*
Fluconazole	149.7 ± 28.6	4.57 ± 2.32
Fluconazole + hypoxia	479.9 ± 54.7*^†^	6.37 ± 2.49

Values represent the mean ± SD of triplicates

*TG* triacylglycerol, *SE* sterol esters

* p < 0.05 for the *t* tests compared to the normoxia culture

^†^ p < 0.05 for the *t*-tests compared to fluconazole culture

#### Sphingolipids with long fatty acyl moieties increase upon hypoxia

**Fig. 4:**
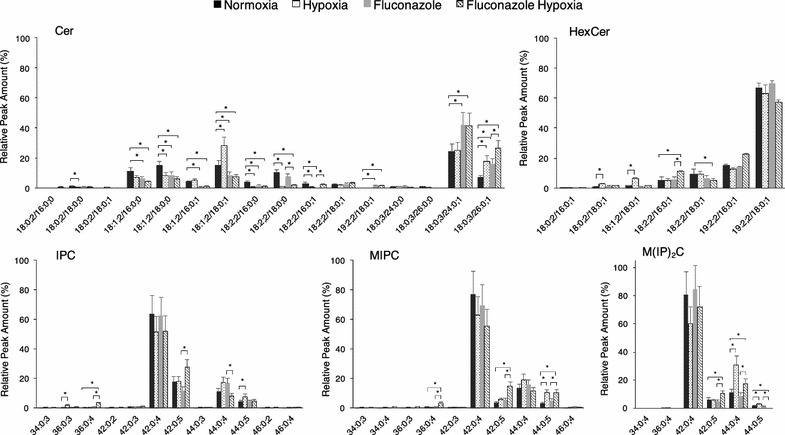
Sphingolipid composition. Sphingolipid analysis of cells growing under normoxic or hypoxic conditions in the presence or absence of fluconazole. Sphingolipid molecular species of ceramides (Cer), hexosylceramides (HexCer), inositolphosphorylceramides (IPC), mannosyl-inositolphosphorylceramides (MIPC) and mannosyl-diinositolphosphorylceramides (M(IP)_2_C) are shown. Species are expressed as long-chain-base/fatty acyl. LCB and fatty acyls are expressed as number of carbons: number of C–C double bonds; number of hydroxyl groups. *p < 0.05 for the *t* tests

Limited availability of oxygen also caused significant changes on the sphingolipid content of *P. pastoris*. Sphingolipids, apart from their function defining membrane structure, associate with ergosterol to form microdomains (“lipid-rafts”), and also play a role as second messengers [66]. The sphingolipid pathway was highly upregulated under hypoxic conditions, as many of the reactions require oxygen (Fig. 1). This was further verified by ddPCR transcriptional analysis of the *SUR2* gene, which appeared to be fourfold upregulated in Fab2F5-producing cells growing in hypoxia. The relative amount of some sphingolipid species was altered under hypoxic conditions (Fig. 4). Specifically, amounts of ceramides with di- and tri-unsaturated long chain base moieties (i.e. 18:2;2, *x*:*y*;*z* denotes a sphingobase or a fatty acid with *x* carbons and *y* double bonds and *z* hydroxyl groups) decreased, which is consistent with the previously mentioned observation that fewer polyunsaturated fatty acids were present under hypoxia. An increase of ceramides carrying methylated moieties was also observed. Notably, the level of ceramide 18:0;3/26:0;1 doubled even though the elongation complex responsible for the synthesis of very long chain fatty acids (*FEN1, SUR4*, *TSC13*) [67], was not upregulated under hypoxia. Hypoxia had also an effect on inositol containing sphingolipids, reducing relative amounts of C42 (i.e. containing C24 fatty acids) species and favoring the content of C44 species (i.e. containing C26 fatty acids). These results may suggest that increasing sphingolipid species with longer fatty acid chains present in membranes under hypoxic conditions could be the way cells adapt to ergosterol and inositol depletion under the culture conditions. It is known that lipid rafts serve as sorting platforms for proteins destined to the cell surface and are involved in cell trafficking [26, 68]. Thus, changes in the sphingolipid and ergosterol content forming these lipid rafts could alter membrane properties, and eventually determine the beneficial effect on Fab secretion.

### Fluconazole treatment reduces the ergosterol content but results in a lipid profile different from the hypoxic condition

Fluconazole is an azole antifungal agent that blocks the ergosterol biosynthesis pathway by inhibiting the Erg11p activity resulting in ergosterol depletion [69]. Fluconazole treatment was used to reduce the ergosterol level, aiming to mimic the effect caused by hypoxia as previously reported [32]. Fluconazole treated cells contained low relative amounts of monounsaturated fatty acids from hypoxic cells (Fig. 2). They also exhibited a significant increase in the relative amounts of PC and PS (Fig. 3). The reduction of the ergosterol content was similar to cells cultured under hypoxia (Table 3), and resulted in the accumulation of ergosterol precursors such as lanosterol. Accumulation of lanosterol and other sterol precursors in cells treated with fluconazole has been previously reported for other yeasts [70, 71]. The sphingolipid content of fluconazole treated cells was characterized by reduced amounts of ceramides species containing dihydrosphingosine and a significant increase of relative levels of ceramides species comprised of phytosphingosine and C24-C26 fatty acyls (Fig. 4). Additionally, the TG content (Table 4) increased in fluconazole treated cells to similar levels as in hypoxic conditions.

**Fig. 5:**
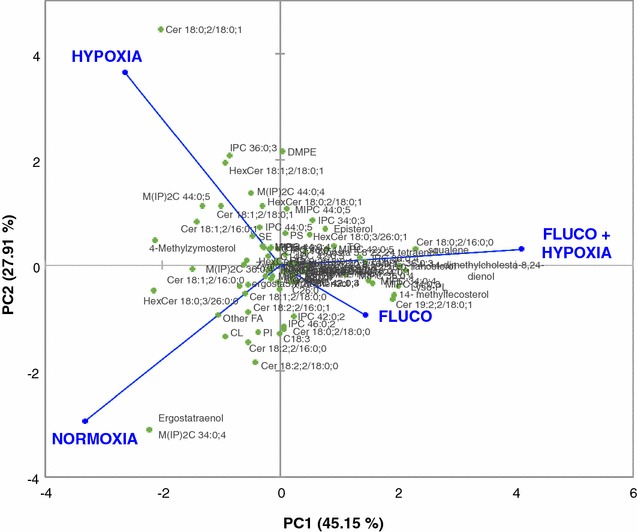
Principal component analysis (PCA) of lipidomic data. Principal component analysis of the lipidome data in a biplot of components one and two. The *biplot* shows lipidomic data (scores) as labelled dots and treatment effect (loadings) as vectors. Vectors that are close together are highly correlated in terms of the observed lipidomic content, while vectors that are orthogonal are poorly correlated. PC1 correlates well with the change due to fluconazole treatment, whereas PC2 appears to be correlated with the change in oxygen conditions

Although both fluconazole treatment and hypoxic cultivation conditions resulted in a marked reduction of ergosterol, lipidome analyses revealed that cells grown under these two conditions displayed significant differences in the profiles of sphingolipids, phospholipids and fatty acids. Interestingly, changes in the lipid composition due to fluconazole treatment increased the specific Fab secretion rate by 1.24-fold, whereas hypoxia lead to a 2.9-fold increase. This observation may be explained by the fact that both fluconazole treatment and hypoxic adaptation although resulting in low ergosterol content seem to provoke pleiotropic and distinct effects (Fig. 5) in the rest of the lipid metabolic network. Moreover, our analyses showed that there was no synergistic effect of fluconazole and hypoxic conditions boosting Fab secretion.

## Conclusions

In the current study, the lipidomic profile of a strain of *P. pastoris* producing a recombinant protein under normoxic and reduced oxygen availability (hypoxia) conditions has been studied. Our results demonstrated regulation of lipid metabolism at the global scale during physiological adaptation to limited oxygen availability, yielding new insight on membrane lipid remodeling under hypoxia and its correlation with improved protein secretion. Based on the results, we postulate that the observed reduction of ergosterol and inositol levels (PI) in cells grown under hypoxia led to lipid stress sensed by UPR. The cellular response on the lipid content included increased storage associated TG species, changes in the PS level and in sphingolipid species.

The results presented here reveal a positive correlation between reduced ergosterol levels and recombinant protein secretion in cells growing under hypoxia and treated with fluconazole. Moreover, our results indicate further changes in the cellular lipid content as a result from the different culture conditions, such as reduction of the ergosterol content accompanied by an increment of TG, reduction of PI levels and changes on sphingolipid content, all of them being positively correlated with increased protein secretion. In this context, recent studies in our laboratory show that disruption of specific genes (e.g. *SUR2*) encoding for sphingolipids species result in significantly increased recombinant protein secretion levels [72], thereby confirming the interplay between membrane lipid metabolism and protein secretion.

In addition, activation of UPR under hypoxic conditions reflects an important interplay between lipid metabolism and protein secretion processes. Importantly, UPR induction by hypoxia—previously observed in both a reference and a Fab3H6-producing strain [23]—does not seem to be strain-specific, as it has also been observed in this study with the Fab2F5-producing strain. Importantly, Gasser and co-workers [73] observed that Fab2F5 overexpression led to the induction of UPR marker genes, although not to the same magnitude as overexpression of the UPR transcription factor Hac1p from *S. cerevisiae*. Also, they were able to improve Fab2F5 production by *HAC1* overexpression (1.3-fold) and *PDI* overexpression (1.9-fold) [74], already pointing at some degree of limitation in secretion taking place in this strain. Therefore, our results indicate that hypoxia was able to increase further transcriptional levels of UPR marker genes. Future studies should allow us to challenge the system for hypoxic growth under even stronger secretory-limiting conditions (e.g. with strains containing different dosages of Fab2F5 expressing cassettes), providing a model to gain new insights on the mechanism underlying the hypoxic effect on protein secretion.

## Methods

### Strain

A *P. pastoris* X-33 strain expressing the light and heavy chain of the human Fab 2F5 antibody fragment was used in this study. The antibody fragment was expressed under the constitutive *GAP* promoter and with the *S. cerevisiae α*-mating factor signal sequence for secretion. The construction of the *P. pastoris* X-33/pGAPZαA-Fab2F5 strain has been previously described [74], and shown to contain multiple copies of the expression cassette (B. Gasser, BOKU, personal communication).

### Chemostat cultivation

Chemostat cultivations were performed in a 2-L Biostat B bench-top bioreactor (Braun Biotech, Melsungen, Germany) at a working volume of 1 L. Cells were grown under glucose-limited conditions at a constant dilution rate (D) of 0.1 ± 0.01 h^−1^ using a peristaltic pump (Ismatec, IDEX Health & Science, Germany) to control the feeding. Cultivations were performed using the batch and chemostat medium compositions detailed elsewhere [75], with minor differences detailed below. 50 g glucose, 1 mL biotin (0.2 g L^−1^), 1.6 mL PTM1 trace salts stock solution [75], and 0.2 mL of antifoam Glanapon 2000 (Bussetti & Co GmbH, Vienna, Austria) were added per liter of chemostat medium. Culture conditions were monitored and controlled at pH 5.0 by addition of 15% (v/v) ammonium hydroxide, temperature of 25 °C, vessel pressure of 1.2 bars, a total gas flow of 1 vvm and pO_2_ above 20% saturation during the batch phase by controlling the stirring rate up to 900 rpm, while it was kept constant at 700 rpm during the continuous phase. Samples were taken for each physiological steady state condition after five residence times (specifically, at the end of the sixth residence time). Online concentrations of the O_2_ and CO_2_ in the exhaust gas of the bioreactor cultivations were determined after being cooled in a condenser (4 °C), dried with two silica gel columns and subsequently analyzed using specific O_2_ and CO_2_ sensors (BCP-CO_2_ and BCP-O_2_. BlueSens, Germany).

### Hypoxic conditions

Cells were grown in chemostat cultures as described in “High Fab secretion yield is observed in all tested culture conditions”, using different concentrations of oxygen in the inlet gas, ranging from 8.03 to 4.02%. The oxygen supply was adjusted by partially replacing airflow with a flow of N_2_. Biomass, glucose, ethanol and arabitol concentrations were measured in the steady state for each oxygen condition. The desired working hypoxic condition was defined as the lower airflow that permitted a stable cell concentration (i.e. no washout in the bioreactor) while significant amounts of ethanol and arabitol were present in the media, thereby indicating respirofermentative metabolic condition. Based on this preliminary series of chemostats, a mixture of 0.25 L min^−1^ air and 0.75 L min^−1^ of N_2_ in the inlet gas were selected, corresponding to a q_p_ of ethanol and arabitol of 0.434 mmol_EtOH_ ${\text{g}}_{\text{DCW}}^{ - 1}$ h^−1^ and 0.048 mmol_Arab_ ${\text{g}}_{\text{DCW}}^{ - 1}$ h^−1^, respectively. Once having established the hypoxic condition, chemostat cultivations were performed in both normoxic and hypoxic conditions.

### Fluconazole treatment

The optimal amount of fluconazole in relation to cell mass allowing for the maximal protein secretion was established. Cells were cultured in shake flasks for 24 h in the presence of different concentrations of fluconazole. The amount of Fab secreted was related to the ratio of fluconazole per final biomass. A value of 80 µg fluconazole per g_DCW_ turned out to be the optimal adjustment, leading to a 1.5-fold increase in yield of secreted Fab. The volume of fluconazole necessary for chemostat cultures was calculated by scaling up the obtained optimal ratio of fluconazole per biomass. Hence, an initial pulse of 320 µL of a fluconazole stock solution (5 mg mL^−1^) was added to the bioreactor at the end of the batch phase to achieve the working fluconazole concentration of 80 μg g ${\text{g}}_{\text{DCW}}^{ - 1}$. Fluconazole levels were maintained along the chemostat cultivation by adding 2 mg of fluconazole per liter of feeding medium.

### Analytical methods

Biomass concentration of the cultivations was determined as dry cell weight (DCW) using a method described [76]. Determinations were performed in triplicate and the relative standard deviations (RSD) were under 4%. Glucose, glycerol, ethanol, arabitol and organic acids (i.e. citric acid and acetic acid) concentrations were determined by HPLC as described [76]. Determinations were performed in triplicate and the RSD was calculated to be below 1%. Fab 2F5 concentration was measured by ELISA as described previously [32]. Determinations were performed in triplicate, and RSD was about 4%.

### Cell disruption and protein extraction

Cells from the cultures were harvested by centrifugation (4500*g*, 4 °C, 3 min), washed twice in cold PBS (pH 7.0) and disrupted as reported [76]. Briefly, cells were resuspended in ice-cold breaking buffer (PBS, 1 mM phenylmethylsulfonyl fluoride (PMSF)), and mechanically disintegrated (two cycles, 2 kbar, 4 °C) using a Constant Systems One-Shot cell disrupter (Daventry, Northants, UK). Cell numbers were determined by means of flow cytometry. After disruption the cell lysate was clarified by centrifugation (15,000*g*, 4 °C for 30 min). The supernatant was collected as soluble cytosolic fraction. The remaining pellet was resuspended with solubilization buffer (10% (w/v) glycerol, 20 mM HEPES pH 7.0, 100 mM NaCl, 1 mM PMSF [77], 1% (w/v) CHAPS), incubated overnight gently mixing at 4 °C to extract the insoluble protein, and centrifuged (2300*g*, 4 °C, 5 min). The supernatant was collected as the insoluble membrane fraction.

### Lipid analysis

Cell homogenates were obtained and lipids were extracted according to Folch et al. [78]. The obtained amounts for all lipids were related to 1 mg total cell protein. Fatty acid, sterol, non-polar lipid and phospholipid composition of cell homogenates were determined as previously described [33]. Phospholipid determinations were performed in duplicate while the rest of lipid species were determined in triplicate.

Analysis of sphingolipid molecular species was performed by liquid chromatography-mass spectrometry. For lipid extraction, 100 mg frozen aliquots of cell wet pellets were processed as previously described in [33]. Ultra-Performance Liquid Chromatography^®^ (UPLC^®^; Waters Corp., Milford, MA, USA) molecular species separation and chip-based nanoelectrospray ionization (TriVersa Nanomate^®^; Advion, Ithaca, NY, USA) were performed as previously described in [79]. Fungal sphingolipid molecular species were detected with a 4000 QTRAP^®^ tandem mass spectrometer (AB Sciex, Framingham, MA, USA) by monitoring the transitions applied in [33]. RSD of the method was never higher than 20%.

### Transcriptional analysis by droplet digital PCR (ddPCR)

The transcriptional levels of the selected set of marker genes for UPR (*HAC1*, *ERO1* and *PDI1*), ergosterol synthesis (*ERG11* and *ERG25)*, fatty acid metabolism (*OLE1* and *FFA1*) and sphingolipid synthesis (*SUR2*) was determined by ddPCR quantification of mRNA levels from total RNA extracts. To normalize data, the house-keeping gene β-actin (*ACT1*) was selected. For cDNA amplification, a set of primers for the target genes plus *ACT1* were designed (Additional file 1: Table S1).

For each culture condition (normoxic and hypoxic), 5-mL samples were mixed with 2.25 mL of chilled 5% (v/v) phenol solution in absolute ethanol and centrifuged at 16,000*g* for 5 min and 4 °C. Resulting pellets were stored at −80 °C. RNA extraction was performed with the RNeasy MiniKit (Qiagen)-iScriptTM. The cDNA Synthesis kit (Bio-Rad) was used for reverse transcription of RNA. Both procedures were carried out following the manufacturer’s protocol. RNA quality was assessed by measuring the 260/280 nm ratio with Nanodrop 1000 (Thermo Fisher Scientific).

The reaction mix used for ddPCR contained: 11.25 μL of QX200TM ddPCR TM EvaGreen Supermix, 200 nM of forward primer, 100 nM of reverse primer, 2.25 ng of cDNA and Dnase/Rnase-free water up to 22.5 μL as a total reaction volume. Droplet formation was carried out using the Droplet Generator QX200TM and further transferred into a 96-well plate. Reactions were incubated at 95 °C for 10 min, followed by denaturation step at 95 °C for 30 s and an annealing/extension step at 57.4 °C for 1 min for a total of 40 cycles. Droplets were detected using the QX100 Droplet Digital PCR System and the software QuantaSoft v. 1.5.38 (Bio-Rad). Positive droplets were normalized for each sample using actin as housekeeping gene.

Normalized mRNA levels of the marker genes were calculated for each sample in duplicate by calculating the ratio between positive droplets of the marker gene and *ACT1* reactions. Reagents for ddPCR were purchased to Bio-Rad (Hercules, CA, US), whereas primers were synthesized by Biomers (Ulm, Germany).

Droplet digital PCR results are summarized in the Additional file 1: Table S2.

### Statistical analysis

Experimental data obtained from chemostat experiments was verified using standard data consistency and reconciliation procedures [80, 81], under the constraint that the elemental conservation relations are satisfied. For all chemostat cultivations performed, the statistical consistency test was passed at a confidence level of 95%, and consequently there was no indication of gross measurement errors. Principal component analysis (PCA) was performed as described elsewhere [24]. Data are shown as mean ± standard deviation (SD). The statistical significance was estimated by Student’s *t* test (two-tailored, unpaired) with Microsoft’s Excel Analysis ToolPak. A statistically significant difference was considered when the *p* value was lower than 0.05.

## Additional file

**Additional file 1: Table S1.** List of primers used for quantitative transcriptional analysis by ddPCR. **Table S2.** Droplet digital PCR analysis of marker genes transcriptional levels. Relative mRNA levels of marker genes under hypoxia related to the normoxic growth condition. All numbers reflect the hypoxic-to-normoxic ratio between relative gene expressions (fold-changes, FC) to the reference gene actin (*ACT1*) under each growth condition. RSD, Relative Standard Deviation.

## Abbreviations

ER: endoplasmic reticulum
UPR: unfolded-protein response
ERAD: endoplasmic reticulum-associated protein degradation
TG: triacylglycerol
PI: phosphatidylinositol
PS: phosphatidylserine
ddPCR: droplet digital PCR
