# Supplementary material for: The effect of hypoxia on the lipidome of recombinant Pichia pastoris
Source: Microb Cell Fact. 2017 May 19;16:86. doi: 10.1186/s12934-017-0699-4 (PMC5437588; doi:10.1186/s12934-017-0699-4)
Supplement: Supplementary file 1 — Additional file 1: Table S1. List of primers used for quantitative transcriptional analysis by ddPCR. Table S2. Droplet digital PCR analysis of marker genes transcriptional levels. Relative mRNA levels of marker genes under hypoxia related to the normoxic growth condition. All numbers reflect the hypoxic-to-normoxic ratio between relative gene expressions (fold-changes, FC) to the reference gene actin (ACT1) under each growth condition. RSD, Relative Standard Deviation. [file 12934_2017_699_MOESM1_ESM.docx]

**Suplementary Material**

**Table S1. List of primers used for quantitative transcriptional analysis by ddPCR.**

| **Gene group** | **Primer name** | **Primer sequence (5'-3')** |
| --- | --- | --- |
| **Sphingolipid synthesis** | **DES1 F** | GGAGAGCTGTAGAAGAACG |
|  | **DES1 R** | CAGTATGAAGTAACACCGCC |
|  | **SUR2 F** | GAATAGCTCCAGTACAATACG |
|  | **SUR2 R** | CCAGCCATACCCTCCAAC |
| **Ergosterol synthesis** | **ERG11 F** | GATGGTAGCAAGATGACCG |
|  | **ERG11 R** | TCCTGCAGCTCTGGTTTC |
|  | **ERG25 F** | CAATGACACCCTTGCCAC |
|  | **ERG25 R** | CCCACTGCTCCTTGTTG |
| **UPR** | **HAC1 F** | CATTACAGCAGGCTCCATC |
|  | **HAC1 R** | GTCAACTGATATGTGCCAAC |
|  | **ERO1 F** | CCTGTCATTGTATAACAGCG |
|  | **ERO1 R** | GTGTTGACCAGTTCCACC |
|  | **PDI F** | CTCAGCATTCTTGGCGTCG |
|  | **PDI R** | GCTTAGAGGCTGGGAGTTC |
| **FFAA**  **metabolism** | **FAA1 F** | CTGCTTAGCCGTCTCCAG |
|  | **FAA1 R** | CCAAACCAGTAGCAATCGCT |
|  | **OLE1 F** | GGAAGTTGTGGTATCCCTC |
|  | **OLE1 R** | CTGTGTTAACTCTCTTGCTC |
| **House-keeping** | **ACT1 F** | TGTCCGGTGGTACTACTATGTTCC |
|  | **ACT1 R** | GATTCGTCGTACTCTTGCTTTGA |

**Table S2**: **Droplet digital PCR analysis of marker genes transcriptional levels**

Relative mRNA levels of marker genes under hypoxia related to the normoxic growth condition. All numbers reflect the hypoxic-to-normoxic ratio between relative gene expressions (fold-changes, FC) to the reference gene actin (*ACT1*) under each growth condition. RSD, Relative Standard Deviation.

| **Gene group** | **Gene** | **Relative transcription change (FC)** | **RSD (%)** |
| --- | --- | --- | --- |
| Sphingolipids synthesis | *SUR2* | 4.114 ± 1.274 | 30.98 |
| Ergosterol- synthesis | *ERG11* | 2.658 ± 0.450 | 16.93 |
|  | *ERG25* | 2.104 ± 0.535 | 25.41 |
| UPR-related genes | *HAC1* | 2.995 ± 1.163 | 38.83 |
|  | *ERO1* | 2.505 ± 0.556 | 22.20 |
|  | *PDI* | 2.076 ± 0.663 | 30.48 |
| FFAA metabolism | *FFA1* | 0.701 ± 0.161 | 22.92 |
|  | *OLE1* | 1.453 ± 0.315 | 21.67 |
